# Increased Nucleosomes and Neutrophil Activation Link to Disease Progression in Patients with Scrub Typhus but Not Murine Typhus in Laos

**DOI:** 10.1371/journal.pntd.0003990

**Published:** 2015-08-28

**Authors:** Daniel H. Paris, Femke Stephan, Ingrid Bulder, Diana Wouters, Tom van der Poll, Paul N. Newton, Nicholas P. J. Day, Sacha Zeerleder

**Affiliations:** 1 Mahidol-Oxford Tropical Medicine Research Unit, Faculty of Tropical Medicine, Mahidol University, Bangkok, Thailand; 2 Centre for Tropical Medicine & Global Health, Nuffield Department of Medicine, University of Oxford, Oxford, United Kingdom; 3 Lao-Oxford-Mahosot Hospital-Wellcome Trust Research Unit, Microbiology Laboratory, Mahosot Hospital, Vientiane, Lao PDR; 4 Sanquin Research, Department of Immunopathology, Amsterdam, The Netherlands; 5 Department of Vascular Medicine and Centre for Experimental and Molecular Medicine (CEMM), Academic Medical Centre, University of Amsterdam, Amsterdam, The Netherlands; 6 Department of Hematology, Academic Medical Centre, Amsterdam, The Netherlands; University of Tennessee, UNITED STATES

## Abstract

Cell-mediated immunity is essential in protection against rickettsial illnesses, but the role of neutrophils in these intracellular vasculotropic infections remains unclear. This study analyzed the plasma levels of nucleosomes, FSAP-activation (nucleosome-releasing factor), and neutrophil activation, as evidenced by neutrophil-elastase (ELA) complexes, in sympatric Lao patients with scrub typhus and murine typhus. In acute scrub typhus elevated nucleosome levels correlated with lower GCS scores, raised respiratory rate, jaundice and impaired liver function, whereas neutrophil activation correlated with fibrinolysis and high IL-8 plasma levels, a recently identified predictor of severe disease and mortality. Nucleosome and ELA complex levels were associated with a 4.8-fold and 4-fold increased risk of developing severe scrub typhus, beyond cut off values of 1,040 U/ml for nucleosomes and 275 U/ml for ELA complexes respectively. In murine typhus, nucleosome levels associated with pro-inflammatory cytokines and the duration of illness, while ELA complexes correlated strongly with inflammation markers, jaundice and increased respiratory rates. This study found strong correlations between circulating nucleosomes and neutrophil activation in patients with scrub typhus, but not murine typhus, providing indirect evidence that nucleosomes could originate from neutrophil extracellular trap (NET) degradation. High circulating plasma nucleosomes and ELA complexes represent independent risk factors for developing severe complications in scrub typhus. As nucleosomes and histones exposed on NETs are highly cytotoxic to endothelial cells and are strongly pro-coagulant, neutrophil-derived nucleosomes could contribute to vascular damage, the pro-coagulant state and exacerbation of disease in scrub typhus, thus indicating a detrimental role of neutrophil activation. The data suggest that increased neutrophil activation relates to disease progression and severe complications, and increased plasma levels of nucleosomes and ELA complexes represent independent risk factors for developing severe scrub typhus.

## Author Summary

Tropical rickettsial illnesses, especially scrub typhus and murine typhus, are increasingly recognized as a leading cause of treatable undifferentiated febrile illness in Asia, but remain severely neglected and under appreciated diseases in many areas. In this study we investigated the relationship of markers of neutrophil activation and cell death with disease severity in patients with acute scrub typhus and murine typhus in Laos. These easily measurable circulating markers were associated with a 4 to 5-fold increased risk of developing severe clinical disease manifestations in scrub typhus and represent independent predictors of severe disease, and possibly death. We also found strong correlations between circulating markers of cell death and neutrophil activation in patients with scrub typhus, but not murine typhus, providing indirect evidence that neutrophil extracellular traps could contribute to the vascular damage and pro-coagulant state leading to exacerbation of disease in scrub typhus, thus indicating a detrimental role of neutrophil activation. The data suggest that increased neutrophil activation relates to disease progression and severe complications, and increased plasma levels of nucleosomes and ELA complexes represent independent risk factors for developing severe scrub typhus.

## Introduction

Typhus-like illnesses, represented by rickettsioses, leptospirosis, dengue and typhoid, pose a significant challenge to tropical infectious disease clinicians due to their non-specific clinical presentations and difficulties in laboratory diagnosis. Recent studies have revealed tropical rickettsioses as leading causes of treatable fevers in Southeast Asia [1–4]. Scrub typhus, caused by infection with *Orientia tsutsugamushi*, and murine typhus, caused by *Rickettsia typhi*, are the predominant species infecting humans in Asia, and probably represent the most frequent, neglected, severe but easily treatable diseases in the world [5,6].

The pathophysiology of scrub typhus and murine typhus in humans remains poorly understood. Mouse models and human post-mortem samples point towards the endothelium as the target of late-stage infection [7–9]. In murine typhus the pathobiology is vasculitic, with *R*. *typhi* targeting the endothelium [9,10]. Recent human *ex vivo* data have revealed differences in endothelial host-pathogen interactions of scrub typhus and murine typhus, based on soluble adhesion molecules, coagulation and inflammation profiles [11,12]. Histopathological studies of eschar skin biopsies have shown an early polymorphonuclear neutrophil (PMN) response in the upper dermis of the eschar, whereas deeper in the dermis the PMNs mix with the predominant mononuclear cell infiltrates, where *O*. *tsutsugamushi* localize mainly within antigen-presenting cells (APCs) [13]. However, *O*. *tsutsugamushi* when phagocytosed by PMNs *in vitro* can escape phagolysosomal fusion and localize freely in the cytoplasm [14]. Neutrophilia is a common finding in patients with scrub typhus and PMNs can be found in perivascular infiltrates of affected organs and cerebrospinal fluid in severe disease [11,15]. Recently, IL-8, which promotes migration of neutrophils to infection sites and a major neutrophil-activating factor, was associated with scrub typhus disease severity [16,17].

The evidence of PMNs playing a role in the host defense against murine typhus is very limited. Clinical reports rarely describe neutrophilia and profiles of soluble adhesion molecules in patients with acute murine typhus suggest endothelial rather than leucocyte activation [11,18]. However, PMNs have been described in the perivascular infiltrates and portal triads of endothelial-tropic spotted fever rickettsiosis, with similar pathogenic mechanisms to *R*. *typhi* infections [19]. Tissue anoxia, metabolic disturbances secondary to vasculitic changes and the host immune response contribute to the pathology of typhus, raising questions about the role of PMNs in the pathogenesis of severe typhus. Basically, PMNs eliminate pathogens by phagocytosis followed by degradation of the pathogens in phagosomes by the NADPH-oxygenase machinery as well as by antibacterial neutrophilic proteins and proteases. Neutrophil degranulation with release of contents of neutrophilic granules to the extracellular milieu can eliminate extracellular pathogens, but can also result in collateral damage of endogenous structures, e.g. endothelial or parenchymal cells. Recent publications have demonstrated PMNs can also form neutrophil extracellular traps (NETs) [20]. NETs are regarded as a form of innate immune response that bind and kill microorganisms, and prevent their dissemination. During NET formation, DNA and DNA-binding proteins are extruded from neutrophils exposing a mesh consisting of nucleosomes, histones and neutrophil proteases such as elastase. Nucleosomes consist of a core octamer with 2 copies of histones H2A, H2B, H3, and H4, around which a helical DNA chain of 146 base pairs is wrapped [21]. They can be actively released by factor VII–activating protease (FSAP) from apoptotic cells and in concert with DNAse 1 from necrotic cells [22,23]. Nucleosomes and histones exposed on these NETs have been reported to be highly cytotoxic to endothelial cells *in vitro* and are strongly pro-coagulant [24–26]. Concentrations of circulating nucleosomes have been associated with mortality and correlated with markers of coagulation, inflammation and neutrophil activation in sepsis [21,27]. Moreover, circulating nucleosomes together with circulating markers for neutrophil activation have been reported to be robust markers to measure NET formation in the circulation [25,28].

PMNs are more important in the host defense of some typhus-like illnesses, i.e. leptospirosis and typhoid than others i.e. dengue. To date, there are no data on PMN activation in scrub typhus and murine typhus. Therefore, we measured levels of circulating nucleosomes and systemic neutrophil activation as evidenced by released neutrophil elastase complexed with its natural inhibitor α_1_-antitrypsin (ELA complexes) as well as FSAP activation in form of FSAP- α_2_-antiplasmin (FSAP-AP) complexes in plasma of patients with confirmed scrub typhus and murine typhus, respectively

## Materials and Methods

### Study population

A total of 248 non-pregnant patients with clinical suspicion of scrub typhus (ST) or murine typhus (MT) were prospectively recruited between Feb 2005 and Dec 2007 at Mahosot Hospital, Vientiane, Lao PDR. Of these, patients with paired positive dynamic serology were randomly selected (ST n = 65, MT n = 64) for measuring plasma levels of nucleosomes, human neutrophil elastase- α_1_-antitrypsin complexes (ELA) and FSAP- α_2_-antiplasmin (FSAP-AP) complexes. Healthy controls (total n = 47) consisted of Dutch (n = 7) and recent Lao blood donors (n = 40).

### Ethics statement

The study was approved by the National Ethics Committee For Health Research, Ministry of Public Health, Lao PDR, and the Oxford Tropical Research Ethics Committee, UK. All patients gave written informed consent prior to sample collection. Minors were participants in this study, and a parent or guardian of any child participant provided written informed consent on their behalf.

### Serological diagnosis

The definitive diagnoses of scrub typhus and murine typhus were based on ≥ 4 fold dynamic rise in IgM and IgG IFA titers for paired serum samples, which represents the current serological gold standard [29]. Slides prepared and standardized by the Australian Rickettsial Reference Laboratory served for anti-*O*. *tsutsugamushi* antibody detection (using pooled Karp, Kato, Gilliam antigens) and anti-*R*. *typhi* antibody detection (*R*. *typhi* Wilmington strain antigens).

### Molecular diagnosis

Bacteremic patients on admission, were identified by realtime PCR, targeting the *groEL* gene for scrub typhus [30] and the *ompB* gene for murine typhus [31], as previously described with modification of the endpoint visualisation by intercalating SYBR green [32]. DNA templates were extracted from 200 μL of Buffy coat collected from EDTA-treated full blood samples (Qiagen Mini Blood kit, Qiagen, Germantown, MD, USA).

### Nucleosome ELISA

Nucleosome plasma levels were measured with a quantitative ELISA assay as described [21,33]. Briefly, a monoclonal antibody (CLB-ANA/60) reacting with histone 3 captured the plasma nucleosomes, which were then detected by monoclonal antibody (CLB-ANA/58 F(ab)2) after binding to complexes of histone 2A, histone 2B and dsDNA (CLB, Amsterdam, the Netherlands).

### FSAP-α_2_-antiplasmin (FSAP-AP) complex ELISA

FSAP-AP complexes in plasma were quantitated as described [22]. Briefly, a mouse monoclonal antibody against AP (AAP-20) was used for catching the FSAP-AP complexes and an antibody targeting the light chain of FSAP (anti-FSAP-4) was used for detection.

### Elastase-α_1_-antitrypsin complex ELISA

Human neutrophil elastase-α_1_-antitrypsin (ELA) complexes were measured by an ELISA as described earlier [34,35]. Briefly, as a catching and detecting antibody a polyclonal rabbit anti-human neutrophil elastase antibody (1.5 μg/ml; Sanquin, Amsterdam, The Netherlands) and a biotinylated monoclonal anti-α_1_-antitrypsin antibody (1 μg/ml) has been used, respectively.

### Markers of coagulation, fibrinolysis, inflammation and endothelium activation

Plasma concentrations of coagulation parameters: Thrombin-antithrombin (TAT) complexes, soluble tissue factor (sTF), tissue- type plasminogen activator (tPA), soluble thrombomodulin (sTM) and von Willebrand factor (VWF) were measured by commercially available ELISAs; Antithrombin (AT), plasminogen activator (PA) activity and plasminogen activator inhibitor-1 (PAI-1) activity were measured with automated amidolytic techniques; Protein C (PC) activity was determined with an amidolytic assay using chromogenic substrate S2366 (Chromogenix, Milan, Italy) as described [12]. Plasma concentrations of TNFα, interleukin (IL)-1β, IL-6, IL-8, IL-10 and IL-12 were measured as previously described [12].

### Skin biopsies

Skin biopsies of eschars were performed with sterile disposable 3 mm circular punch biopsies (Stiefel Laboratories Inc., Offenbach, Germany) to sample the necrotic edge with perifocal inflamed skin, after local anesthesia with 1% lidocaine. Biopsies in this patient cohort were performed for co-localisation and pathophysiology studies. For this study, consenting patients (n = 2) had a biopsy taken, which was fixed in 10% formalin and processed later into paraffin blocks.

### Statistical analysis

Results are reported as medians and interquartile ranges (IQR) unless noted otherwise. Patient data were compared between groups using the Kruskal-Wallis test or Mann-Whitney U tests. Correlations between variables were assessed using Spearman’s rank correlation and Pearson’s product correlation where appropriate, corrected for multiple testing with the Bonferroni method. Receiver operating characteristic (ROC) analysis provided the selection of optimal cut off points for circulating nucleosomes and EA complexes between controls and cases, as well as between severe and non-severe disease. The associations between circulating nucleosomes and EA complexes and the association of developing complicated disease were explored by means of logistic regression analysis and are expressed as odds ratios (OR) with corresponding 95%CIs. Statistical significance was set at p<0.05. All statistical analyses were calculated using Stata/MP 11.0 (Stata Corp., College Station, Texas, USA).

## Results

### Patient characteristics

Patients in both disease groups did not differ significantly by age, gender, days of fever and symptoms prior to admission (Table 1). The presence of an eschar, lymphadenopathy and muco-cutaneous hemorrhages were significantly associated with scrub typhus patients (all p-values <0.0001). All typhus patients survived to discharge. Laboratory parameters outside the normal range that differed between the two forms of typhus were plasma albumin, C-reactive protein (CRP) and lactate dehydrogenase (Table 1). The median (IQR) bacterial loads differed significantly between bacteremic patient subgroups with 2,100 copies/mL (800–4,500) and 700 copies/mL (400–1100) in full blood, for scrub typhus and murine typhus patients respectively (p = 0.0001).

**Table 1:** Demographic, clinical and laboratory characteristics of all patients in this study.

Parameter	Unit	Scrub Typhus	Murine Typhus	p-value
Age*	Years	27 (5–76)	29 (9–82)	0.15
Days of fever^†^	Days	9 [7–11]	8 [7–9]	0.30
ADM-FUP^‡^	Days	6 [3–7]	5 [3–7]	0.74
Eschar	*NA*	28/65 (43%)	0/58 (0%)	***0*.*0001***
Skin rash	*NA*	10/65 (15%)	10/61 (16%)	0.88
Lymphadenopathy^§^	*NA*	39/65 (60%)	6/62 (10%)	***0*.*0001***
Hemorrhage^¶^	*NA*	28/65 (42%)	5/64 (8%)	***0*.*0001***
Hearing loss	*NA*	8/38 (21%)	1/18 (6%)	0.14
GCS	Score	15 [7–15]	15 [15–15]	0.09
WBC	x10^3^/mL	9.7 [6.9–12.8]	8.1 [6.8–10.9]	0.13
Neutrophils	(%) WBC	68 [58–77]	65 [56–71]	0.06
	Abs count	6,4 [4,2,0]	4,7 [3,8–6,8]	***0*.*004***
Lymphocytes	(%) WBC	30 [20–40]	34 [26–41]	0.22
Monocytes	(%) WBC	6.3 [0–9.3]	2 [0.3–4]	0.63
Platelets	1,000 / mL	200 [170–220]	200 [170–210]	0.48
Sodium	mmol/L	137 [132–141]	146 [139–151]	***0*.*0001***
Creatinine	μmol/L	88.4 [62–106]	106 [88–128]	***0*.*0012***
Albumin	g/L	32 [27–37]	39 [33–41]	***0*.*0001***
Blood urea nitrogen	mmol/L	3.6 [2.9–5]	3.2 [2.5–4.6]	0.18
Aspartate transaminase	U/L	91 [63–148]	75 [47–108]	0.07
C-reactive protein	U/L	74 [48–131]	50 [26–112]	***0*.*035***
Lactate dehydrogenase	U/L	564 [464–665]	431 [309–551]	***0*.*0002***

*Abbreviations*: *NA*: *not available; ADM-FUP*: *time between admission and follow-up; GCS*: *Glasgow coma scale; WBC*: *white blood cell count;*

* *Values in round brackets represent age range*.

† *Number of febrile days prior admission*.

‡ *Admission to follow-up period for cytokines*, *coagulation and biochemistry markers*.

§ *Includes regional and/or generalized lymphadenopathy*.

¶ *Hemorrhage was defined as (muco)-cutaneous petechial and suffusion bleeding*. Comparisons of demographic, clinical, hematological and biochemical parameters for scrub typhus (n = 65), murine typhus (n = 64). Values are depicted as median with interquartile range [IQR]. Significant p-values are depicted in bold italics. Probability-values were calculated by the Kruskal-Wallis equality-of-populations rank test.

### Circulating nucleosomes and neutrophil activation

**Fig 1:**
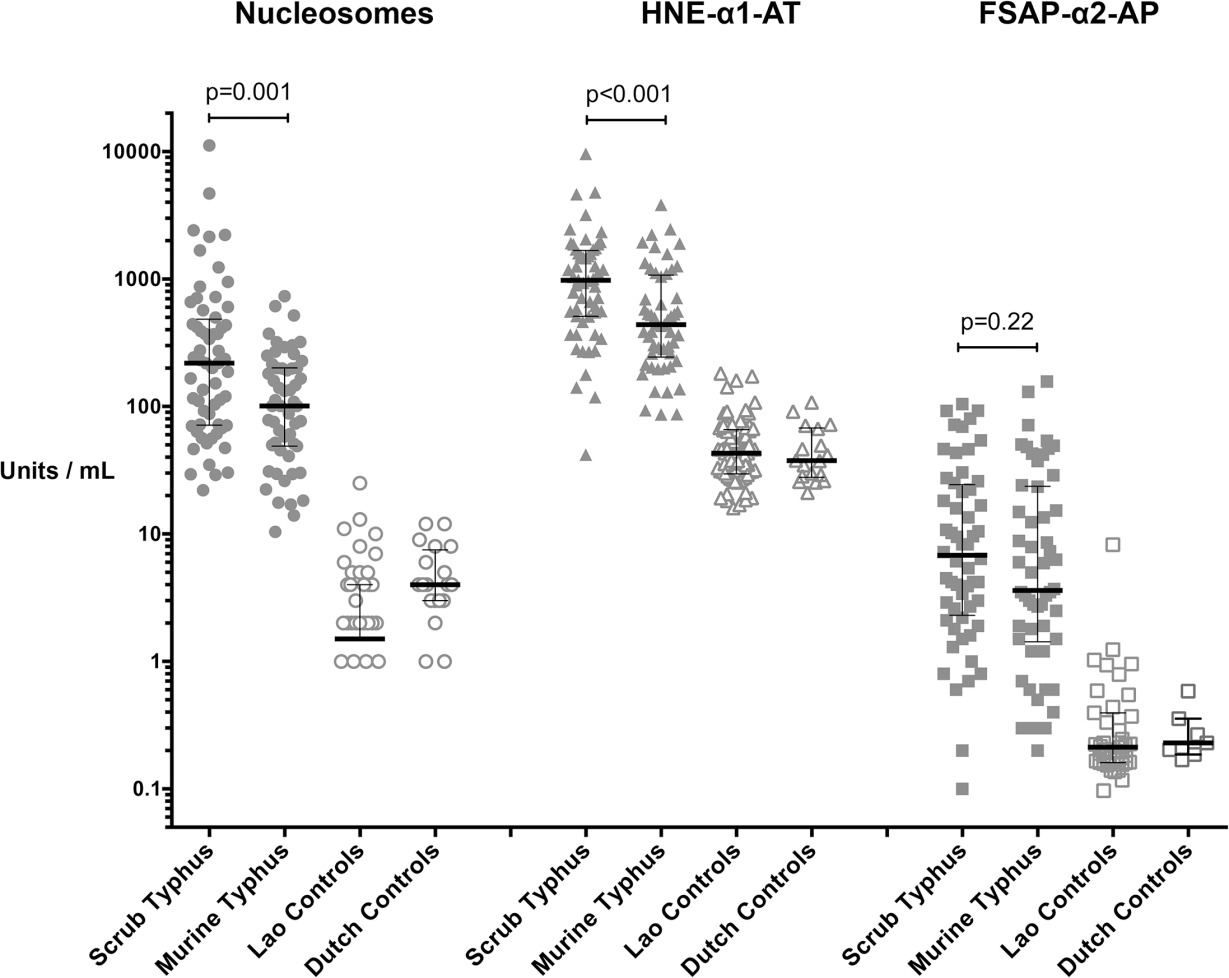
Markers of cell death and neutrophil activation in patients with scrub typhus, murine typhus and controls. Markers of cell death and neutrophil activation. Plasma levels of nucleosomes, human neutrophil elastase (ELA) complexes and Factor VII activating protease anti-plasmin complexes (FSAP) were significantly higher in patients with scrub typhus (ST) and murine typhus (MT) than in Asian (Laos) and Caucasian (Dutch) controls (p-values all *<0*.*0001*). On comparison between ST and MT, the nucleosome and ELA-complex plasma levels were significantly higher in ST than in MT, but not the FSAP levels. Data are expressed as median with interquartile range (whiskers) of admission samples.

The plasma levels of nucleosomes, ELA and FSAP-AP complexes in both scrub typhus and murine typhus were significantly higher than in Dutch and Lao controls (all p-values <0.0001). The plasma levels of nucleosomes and ELA complexes were significantly higher in scrub typhus than in murine typhus (p = 0.001 and p<0.001 respectively, Fig 1). The median (interquartile range) plasma levels for nucleosomes, ELA and FSAP complexes were 219 [72–471], 977 [511–1647], 6.8 [2–24] U/ml for scrub typhus and 101 [49–201], 440 [260–1046], 3.6 [2–24] U/ml for murine typhus and 2 [0–4], 46 [31–65], 0.2 [0.2–0.4] U/ml for Lao controls and 4 [3–7], 38 [28–67], 0.2 [0.2–0.4] U/ml for Dutch controls, respectively.

Plasma levels of circulating nucleosomes and ELA complexes correlated strongly with each other in the pooled typhus patient groups (Spearman correlation coefficient Rho 0.452; p<0.001), but not within pooled or between control groups. This study included two sets of controls to ensure that the background levels of all markers measured did not differ between the local Lao healthy population and European Caucasians. Healthy control values were previously characterized for these assays, with no statistical differences of results in and between the control groups [27,34]. Stratification by disease into scrub typhus versus murine typhus, demonstrated differential profiles: in scrub typhus nucleosomes correlated strongly with ELA complexes (Rho 0.495; p = 0.005), whereas in patients with murine typhus nucleosomes correlated strongly with FSAP complexes (Rho 0.492; p = 0.01) (Table 2).

**Table 2:** Plasma nucleosomes levels and neutrophil elastase (ELA) complexes correlate in typhus patients. The first column lists markers of demographic, clinical and laboratory features of patients with acute scrub typhus or murine typhus, which correlated with plasma nucleosome and ELA complex levels. Correlation coefficients (Rho) and p-values are reported for Rho values >0.30 or p values <0.05.

	Nucleosomes		ELA complexes	
	*Rho*	*P value* ^*^	*Rho*	*P value* ^*^
**Scrub Typhus**				
Nucleosomes	1	—	0.50	***<0*.*001***
FSAP-AP	0.32	0.50	0.48	***0*.*003***
ELA complexes	0.50	***<0*.*001***	1	—
GCS	0.56	***<0*.*001***	—	*—*
Respiratory Rate	0.60	***<0*.*001***	—	*—*
Jaundice	0.52	***<0*.*001***	—	*—*
Bilirubin direct	0.30	***0*.*021***	—	*—*
LDH	0.49	***0*.*008***	—	*—*
ALT	0.28	***0*.*03***	0.26	***0*.*05***
PAA	*—*	*—*	0.52	***<0*.*001***
IL-8	*—*	*—*	0.33	***<0*.*02***
**Murine Typhus**				
Nucleosomes	1	—	0.29	1
FSAP-AP	0.49	***0*.*013***	0.34	0.25
ELA complexes	0.29	1	1	—
Days ill	-0.32	***0*.*02***	—	—
CRP	0.36	***0*.*012***	—	—
IL-6	0.54	***<0*.*001***	—	—
IL-8	0.60	***<0*.*001***	—	*—*
ALT	0.32	***0*.*019***	—	—
IL-10	0.37	***0*.*006***	0.30	***0*.*04***
RR	—	—	0.35	***0*.*01***
Jaundice	—	—	0.32	***0*.*02***
Albumin	—	—	0.35	***0*.*01***
TNFα	—	—	0.43	***0*.*003***

*Note*: *Spearman Rank test or Pearson Correlation*, *where appropriate*, *Bonferroni correction for multiple testing*. *Only values with Rho values >0*.*30 or p values <0*.*05 were included*. *Significant p values in bold italic*. *Data log transformed where appropriate*.

### PMN counts in scrub typhus and murine typhus

Neutrophil counts were significantly higher in scrub typhus patients than in murine typhus patients with a median (IQR) of 6,392/mL (4,240–9,005) and 4,674/mL (3,822–6,831), respectively (p = 0.004) (Table 1). When stratified for disease severity, the non-severe scrub typhus patients had a significantly higher neutrophil count than non-severe murine typhus patients (p = 0.001), while neutrophil counts were similar for both diseases in severe disease (p = 0.87).

In scrub typhus, PMN counts correlated (spearman’s Rho, p-value) with plasma levels of IL-6 (Rho = 0.257, p = 0.01), bleeding (Rho = 0.26, p = 0.041) seizure (Rho = 0.371, p = 0.002), confusion (Rho = 0.304, p = 0.015) and GCS (Rho = 0.22, p = 0.023), but not with ELA complex levels (p = 0.314).

In murine typhus, PMN counts correlated well (Spearman’s Rho, p-value) with plasma levels of protein C (Rho = 0.37, p = 0.0089), CRP (Rho = 0.31, p = 0.0026), and the presence of skin rash (Rho = 0.23, p = 0.031), but not with ELA complex levels (p = 0.074).

### High-density neutrophil infiltrates in eschar

**Fig 2:**
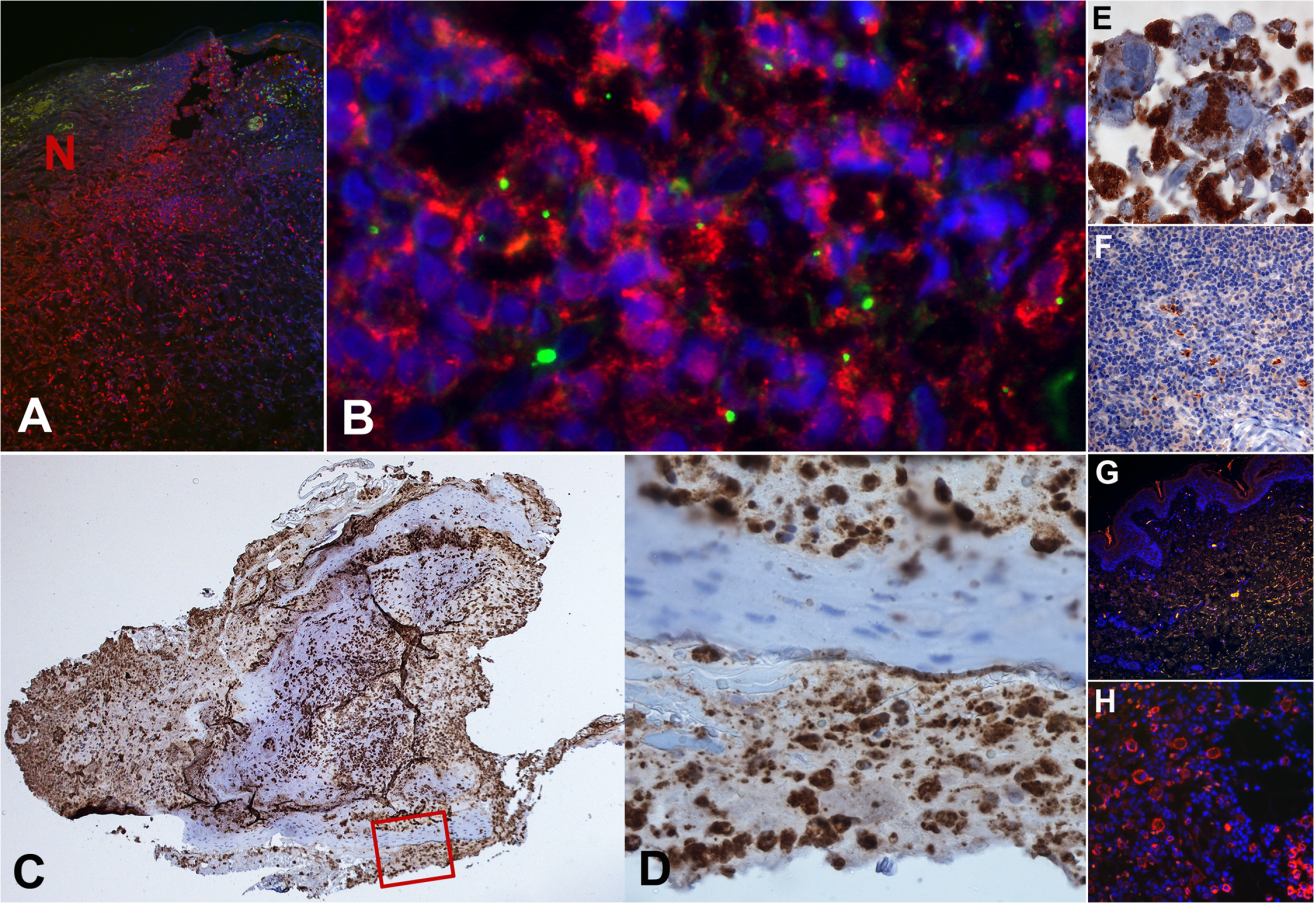
Neutrophils are prominent in the eschars of scrub typhus patients. The necrotic zone of a human eschar biopsy in a patient with scrub typhus is characterized by high-density neutrophil infiltrates with partially karyorrhectic neutrophils. Panel A: The granulocyte-dense zones are predominantly located adjacent to the central necrotic zone (N), at the dermal-epidermal junction and in superficial dermal infiltrates (anti-neutrophil elastase stained red, patient TM2193, magnification x400). Panel B: Immunofluorescence image of a neutrophil rich infiltrate of the upper dermis containing *Orientia tsutsugamushi* stained bright apple green (in-house anti-56kDa Orientia mAb) and neutrophils stained red (anti-CD15 antibody, Abcam By87), patient TM2193, magnification x600). Panels C and D: Immunohistochemical staining of neutrophils in brown, using anti-CD15 antibodies (Abcam By87). Panel D is an enlargement and corresponds to the red square insert, demonstrating the typical granulation of neutrophils. Patient TM 2644, magnification x100, insert x600. Panels E-H: Controls included anti-56kDa Orientia mAb positive control in heavily-infected L929 cells (immunohistochemistry, x600, panel E); anti-CD15 antibody (Abcam By87) positive control in uninfected human tonsil (immunohistochemistry, x400, panel F) and skin (immunofluorescence with anti-CD15 in red, x100, panel G); and anti-56kDa Orientia mAb negative control in chronically inflamed (psoriasis) human skin (immunofluorescence with HLADR in red and anti-56kDa in green, x400, panel F).

*O*. *tsutsugamushi* co-localized with neutrophils in high-density neutrophil infiltrates predominantly located adjacent to the central necrotic zone of the eschar lesion, at the dermal-epidermal junction along the necrotic margin and in superficial dermal infiltrates (Fig 2). *O*. *tsutsugamushi* and partially karyorrhectic neutrophils were also found in high densities embedded in the necrotic crust, providing descriptive evidence that the easily accessible and painlessly detachable necrotic crust represents a useful specimen for molecular diagnostics.

### Severe typhus

To date no validated algorithm for assessing the severity of rickettsial infections is available. In this study severe typhus was defined when the following admission clinical criteria were apparent: a Glasgow Comma Score (GCS) of <15, clinical evidence of meningism or central nervous system involvement and/or a respiratory rate (RR) of >20/min. In total, 27/128 (21%) of scrub typhus and 14/102 (14%) of murine typhus patients had evidence for CNS and/or respiratory tract involvement and/or reduced vigilance at presentation.

**Fig 3:**
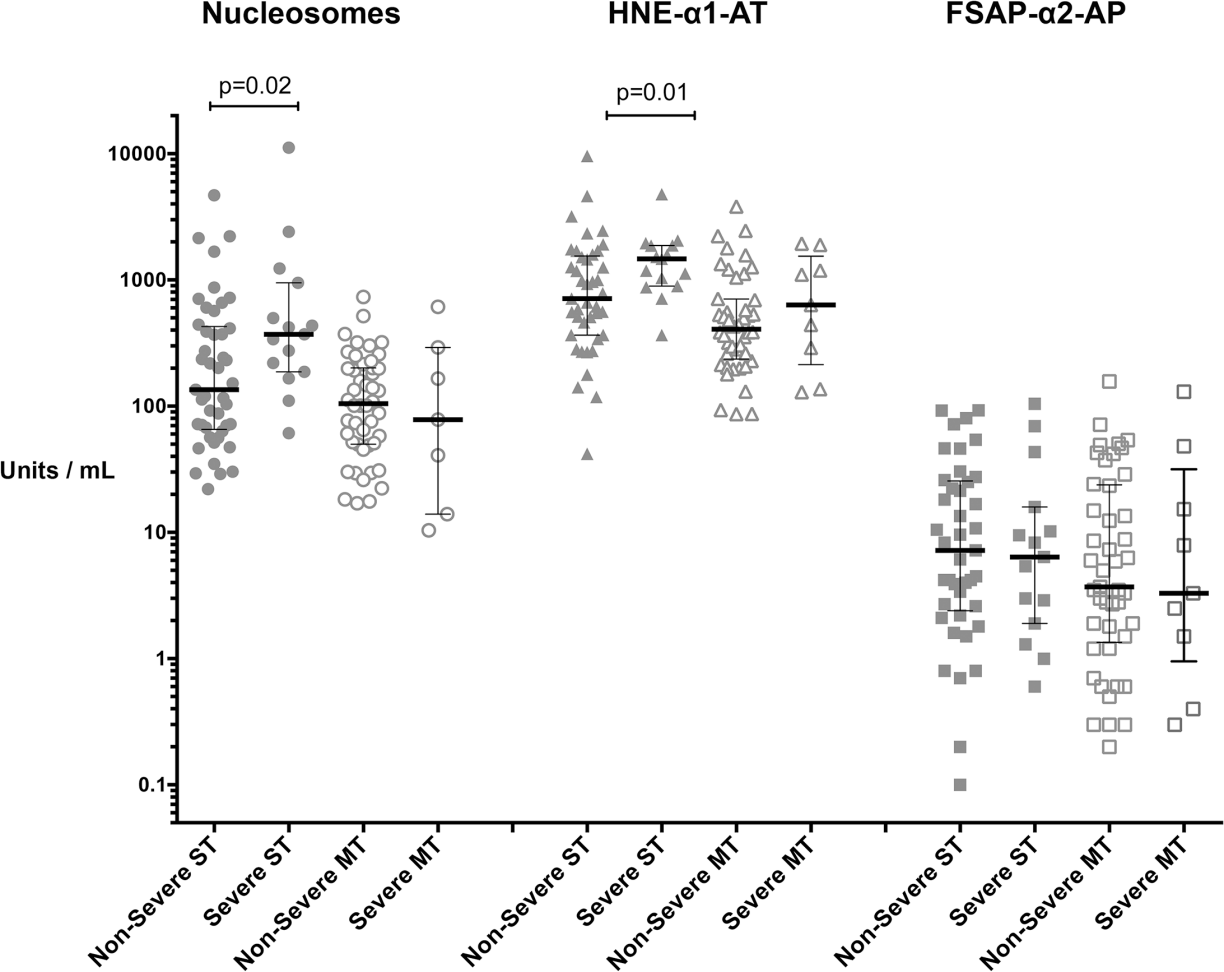
Nucleosomes and neutrophil elastase (ELA) complex plasma levels are raised in severe scrub typhus. Plasma levels of nucleosomes, ELA complexes and FSAP complexes were significantly higher in patients with severe scrub typhus than in non-severe cases (p = 0.02 and p = 0.01 respectively), but not FSAP complexes. Increased disease severity was not reflected by higher levels of nucleosomes or ELA complexes in murine typhus patients, although the p-values of p = 0.06 and p = 0.08, respectively, are suggestive of a trend. Scrub typhus data are bold, while murine typhus data are outlined. Data are expressed as median with inter- quartile range (whiskers) of admission samples.

Median (IQR) plasma levels of nucleosomes and ELA complexes were significantly higher in patients with severe scrub typhus than in non-severe patients (p = 0.02 and p = 0.01, respectively), with 372 U/ml (187–952) versus 136 U/ml (68–414) and 1471 U/ml (893–1876) versus 713 U/ml (367–1515), but FSAP complexes were not (Fig 3). Increased disease severity was not reflected by higher levels of nucleosomes or ELA complexes in murine typhus patients, although the p-values of p = 0.06 and p = 0.08, respectively, are suggestive of a trend (Fig 3).

### Odds of developing severe disease

We performed ROC analysis to calculate the optimal cut off associated with the highest percentages of correct predictions into severe or non-severe groups. The cut off values corresponded to 1,040 U/ml for circulating nucleosomes and 275 U/ml for ELA complexes, respectively. For patients with scrub typhus there was a statistically significant increased risk of developing severe disease, if nucleosomes or ELA complex plasma levels were greater than these cut offs with an OR of 4.0 (95% CI 1.2–13.8; p = 0.028) and 4.8 (95%CI 1.3–17.6; p = 0.019) respectively.

In patients with murine typhus, the cut off points corresponded to 129 U/ml and 14 U/ml for ELA complexes and for nucleosomes, respectively; no significant association was found for these markers and disease severity.

### Relationship to coagulation, inflammation, clinical and laboratory markers

A proportion of enrolled patients had coagulation and inflammation data available for subgroup analyses; for scrub typhus 44/65 (68%) and for murine typhus 44/64 (67%) (Table 2).

Among patients with scrub typhus, elevated nucleosomes correlated with involvement of the CNS, lung and liver, as evidenced by correlations to GCS, respiratory rate and liver function tests. In murine typhus, nucleosomes correlated with pro-inflammatory cytokines, liver transaminases and days of illness, whereas ELA complexes correlated with lung and liver involvement and inflammatory markers (Table 2).

## Discussion

Although the importance of cell-mediated adaptive immune response in host protection against scrub typhus and murine typhus is well established, the role of neutrophils in these intracellular infections remains unclear. Pathophysiological studies of surrogate markers for cell adhesion molecules, coagulation and inflammation including histopathological observations in patients with scrub typhus and murine typhus, have highlighted differential profiles for the two diseases at the hospital admission time point [11–13,17]. In this study we hypothesized that neutrophils could provide a differential contribution to disease severity in these diseases, and measured the plasma levels of nucleosomes, FSAP activation and neutrophil activation in sympatric patients with scrub typhus and murine typhus in Laos. Although raised levels of circulating nucleosomes and systemic neutrophil activation were found in patients with both forms of typhus compared to controls, the increase of both markers was significantly higher in scrub typhus than in murine typhus (Fig 1).

In scrub typhus, neutrophilia was recently described in the acute phase of infection [15], but if neutrophils relate to a beneficial or detrimental role in the human pathogenesis of typhus has not been determined. In this study, neutrophil counts correlated with clinical signs of severe disease in patients with scrub typhus, and were significantly higher than in murine typhus (Table 1). That blood neutrophil counts did not correlate well with ELA complexes in both diseases could suggest that peripheral PMNs reflect the circulating pool, but not the marginal pool and PMNs located in tissue infiltrates, which in these vascular diseases could be substantially larger [36–38]. The immune histological investigation of skin biopsies of inoculation eschars (Fig 2), revealed the presence of prominent neutrophil-rich infiltrates within and adjacent to the necrotic center, as well as in perivascular infiltrates, suggesting that PMNs play a prominent role in the first-line defense against *O*. *tsutsugamushi* [7,13]. Although blood neutrophilia does not reflect the extent of systemic neutrophil activation, the data suggest that neutrophils could contribute more to disease severity in scrub typhus than previously known and that measuring neutrophil activation and nucleosomes may be useful for assessing disease severity in analogy to findings in sepsis patients. This study did not determine if neutrophils contribute to host defense against *O*. *tsutsugamushi*, but as disease severity and disease resolution can be independent of each other, the evidence tends towards a detrimental effect of neutrophil activation, associated with disease progression and complications. Neutrophil activation was raised in murine typhus, but significantly less prominent than in scrub typhus. In murine typhus patients, neutrophil activation did not associate with nucleosomes or disease severity, but an association to disease severity was seen (Table 2).

Nucleosomes are histones and cell-free DNA released upon cell death. The high nucleosome levels in scrub typhus patients showed a strong association with disease severity, which was reflected in lower GCS scores, raised respiratory rates, jaundice and impaired liver function (Table 2). This association was not found in patients of the murine typhus group, where raised nucleosome levels associated with inflammatory cytokines, systemic inflammation and the duration of illness, while ELA complexes correlated strongly with inflammation, jaundice and increased respiratory rates. Even though these markers were not significantly higher in the severe murine typhus group (p-values 0.06 and 0.08 respectively, Fig 3), the findings merit further investigation of measuring nucleosomes and ELA complexes for assessing disease severity in analogy to findings in sepsis patients [21,27].

We found significantly higher bacterial loads for *O*. *tsutsugamushi* than *R*. *typhi*, which is in line with recent findings of higher bacteremic burden during scrub typhus than in murine typhus [39]. The increased circulating biomass of *O*. *tsutsugamushi* could contribute to a more pronounced systemic inflammation and exposure to neutrophils, resulting in marked systemic neutrophil activation, and a pro coagulant and inflammatory state, as reflected by higher nucleosome and neutrophil activation levels [12,40]. These data are congruent with recent reports that demonstrated neutrophil activation and nucleosome levels to correlate with disease severity and fatality in systemic inflammations, such as severe sepsis, septic shock and meningococcal sepsis [21,27].

FSAP circulates as a single-chain molecule in plasma and is activated upon contact with apoptotic and necrotic cells [22,23]. Recently, cell-free histones, RNA and glycosaminoglycans have been reported as FSAP activators as well [41–43]. Activated FSAP induces cleavage of DNA by DNAse resulting in nucleosome release from apoptotic cells and necrotic cells [22,44]. In the absence of any of these enzymes, DNA will not be released, therefore, the local concentration of DNAse, which acts in concert with FSAP to remove DNA from necrotic cells, critically influences nucleosome release as well [45].

In this study we found FSAP activation in both sympatric scrub and murine typhus patients, suggestive of involvement of FSAP in the release of nucleosomes in both diseases. Interestingly, strong correlations with nucleosomes were only observed in murine typhus, but not scrub typhus patients (Table 2). The activators of FSAP have not been identified for these forms of typhus, but our findings may indicate different mechanisms of FSAP activation, derived from potentially different sources of extracellular DNA for scrub and murine typhus. Although, the actual source of circulating nucleosomes remains difficult to establish, in inflammatory diseases nucleosomes are released from both dead hematopoietic and parenchymal cells. The different correlations between nucleosomes, FSAP and neutrophil activation in these intracellular infections suggest distinct cell death patterns occur, but the source of dead cells remains to be determined. If the cellular tropism of *R*. *typhi* and the associated cytotoxic T cell responses providing protective immunity in part by cytotoxicity towards infected cells contribute to nucleosome release remains to be determined [8,46]. However, in scrub typhus the data point towards neutrophils as a potential source of nucleosomes, contributing to disease exacerbation reflected by association to clinical disease severity (Table 2).

The finding of high circulating plasma nucleosomes and ELA complexes as independent risk factors for developing severe complications in scrub typhus suggests that increased neutrophil activation could have a detrimental influence to disease severity, and no evidence points towards elevated nucleosomes and ELA complexes contributing towards resolving infection. In previous studies, nucleosomes and histones exposed on NETs have been reported to be highly cytotoxic to endothelial cells in vitro and are strongly pro-coagulant [24–27,40]. Further, concentrations of circulating nucleosomes have been associated with mortality and correlated with markers of coagulation, inflammation and neutrophil activation in sepsis [21,27].

Scrub typhus is a systematic vasculopathy with pronounced perivascular infiltrates and a procoagulant inflammatory coagulation profile [12]. Upon admission, scrub typhus patients with increased plasma levels of nucleosomes or ELA complexes above the specific cut off values (1,040 U/ml for circulating nucleosomes and 275 U/ml for ELA complexes) were associated with a 4.8-fold and 4-fold increased risk of developing severe disease. Neutrophil activation correlated strongly with nucleosome levels, which suggests that neutrophils contribute to histone circulation, leading to subsequent vascular damage and resulting in a pro-coagulant profile, thus actually contributing to exacerbation of disease. That ELA complexes also correlated with fibrinolysis and high IL-8 plasma levels, a recently identified predictor of severe disease and mortality [17], further corroborates the harmful effect of neutrophil activation.

These results confirm that high levels of circulating nucleosomes and ELA complexes are independent risk factors for developing severe scrub typhus. Studies published by the Wagner group demonstrate that circulating nucleosomes and markers for neutrophil activation are reliable PMN markers associated with NET formation in the circulation [25,28]. By using comparable markers we find strong correlation of nucleosome levels with neutrophil activation in scrub typhus patients suggesting that neutrophils represent—at least in part—the source of nucleosomes in these patients, providing indirect evidence for NET formation. In summary, the data suggest that increased neutrophil activation relates to disease progression and severe complications in scrub typhus, but not murine typhus, and that increased plasma levels of nucleosomes and ELA complexes represent independent risk factors for developing severe scrub typhus.
